# Phenotypic sexual dimorphism is associated with genomic signatures of resolved sexual conflict

**DOI:** 10.1111/mec.15115

**Published:** 2019-06-05

**Authors:** Alison E. Wright, Thea F. Rogers, Matteo Fumagalli, Christopher R. Cooney, Judith E. Mank

**Affiliations:** ^1^ Department of Animal and Plant Sciences University of Sheffield Sheffield UK; ^2^ Department of Life Sciences Imperial College London London UK; ^3^ Department of Genetics, Evolution and Environment University College London London UK; ^4^ Department of Organismal Biology Uppsala University Uppsala Sweden; ^5^ Department of Zoology University of British Columbia Vancouver British Columbia Canada

**Keywords:** molecular evolution, population genetics, sexual conflict, transcriptomics

## Abstract

Intralocus sexual conflict, where an allele benefits one sex at the expense of the other, has an important role in shaping genetic diversity of populations through balancing selection. However, the potential for mating systems to exert balancing selection through sexual conflict on the genome remains unclear. Furthermore, the nature and potential for resolution of sexual conflict across the genome has been hotly debated. To address this, we analysed de novo transcriptomes from six avian species, chosen to reflect the full range of sexual dimorphism and mating systems. Our analyses combine expression and population genomic statistics across reproductive and somatic tissue, with measures of sperm competition and promiscuity. Our results reveal that balancing selection is weakest in the gonad, consistent with the resolution of sexual conflict and evolutionary theory that phenotypic sex differences are associated with lower levels of ongoing conflict. We also demonstrate a clear link between variation in sexual conflict and levels of genetic variation across phylogenetic space in a comparative framework. Our observations suggest that this conflict is short‐lived, and is resolved via the decoupling of male and female gene expression patterns, with important implications for the role of sexual selection in adaptive potential and role of dimorphism in facilitating sex‐specific fitness optima.

## 1 INTRODUCTION

Males and females in many species often have divergent evolutionary interests and are subject to conflicting selection pressures (Andersson, 1994). However, with the exception of the sex chromosomes, the sexes share an identical genome, and this can give rise to intralocus sexual conflict, where an allele benefits one sex at the expense of the other (Parker & Partridge, 1998). This shared genomic architecture is thought to hamper males and females simultaneously evolving towards their respective fitness peaks, and in turn acts as a constraint in the evolution of sexual dimorphism (Mank, 2017; Rowe, Chenoweth, & Agrawal, 2018; Stewart & Rice, 2018).

Recently, studies have used population genomic statistics to detect the signature of sexual conflict across the genome (Cheng & Kirkpatrick, 2016; Dutoit et al., 2018; Lucotte, Laurent, Heyer, Ségurel, & Toupance, 2016; Mank, 2017; Mostafavi et al., 2017; Rowe et al., 2018; Wright et al., 2018). Ongoing sexual conflict can arise from several different factors and these leave distinct population genomic signatures in sequence data (Mank, 2017; Wright et al., 2018). Sexual conflict can result in over‐reproduction, where an allele increases the reproductive fitness of one sex at a cost to the other (Barson, et al. 2015; Lonn et al., 2017). Alternatively, sexual conflict can result when an allele has differential effects on survival between males and females (Czorlich, Aykanat, Erkinaro, Orell, & Primmer, 2018). Both of these scenarios are predicted to produce in elevated genetic diversity and higher Tajima's *D*, a population genomic statistic that estimates the proportion of polymorphic nucleotide sites in a given sequence within a population.

To distinguish between sexual conflict arising over reproduction or survival, it is necessary to employ contrasts with intersexual *F*_ST_ (Lewontin & Krakauer, 1973), which measures divergence in allele frequency between males and females within a generation. As allele frequencies are identical between the sexes at conception, different allele frequencies in male and female adults are assumed to be the result of sexual conflict over survival. Elevated *F*_ST_ can therefore be used to identify alleles that have differential effects on survival parameters, including viability, mortality or predation. By contrasting these two population genomic statistics, it is possible to determine the relative importance of conflict over reproduction, which only leads to increased Tajima's *D*, versus conflict over survival, which leads to elevated Tajima's *D* and intersexual *F*_ST_ (Mank, 2017; Wright et al., 2018).

Population genomic approaches such as these have made it possible to investigate the manifestation of different types of intralocus sexual conflict at the genomic level and the mechanisms by which they can be resolved. In theory, sexual conflict should be most prevalent in genes with similar expression patterns in males and females, where mutational inputs will be manifest in both sexes. Ultimately, sexual conflict is thought to be resolved via the evolution of sex‐biased gene expression (Connallon & Knowles, 2005; Ellegren & Parsch, 2007), which, because of primary expression in one sex or the other, in principle allows for the emergence of male‐ and female‐specific fitness optima (Mank, 2017). However, the exact nature of the relationship between sex‐biased gene expression and resolved sexual conflict has been hotly debated, with some recent studies suggesting that sex‐biased genes are subject to ongoing sexual antagonism (Cheng & Kirkpatrick, 2016; Dutoit et al., 2018). If true, this suggests that sexual conflict can persist even after gene expression diverges between males and females, and is potentially an unrelenting constraint on sex‐specific optima. It would also suggest that, although expressed primarily in one sex, sex‐biased genes function similarly in both males and females, and are therefore not appropriate for studying molecular signatures of sex‐specific selection, as is often done (Ellegren & Parsch, 2007).

Moreover, the signature of balancing selection for sex‐biased genes detected by recent studies is discordant with the rapid molecular evolutionary rates of directional selection (Meiklejohn, Parsch, Ranz, & Hartl, 2003; Pröschel, Zhang, & Parsch, 2006; Zhang, Sturgill, Parisi, Kumar, & Oliver, 2007) and relaxed constraint (Dapper & Wade, 2016; Gershoni & Pietrokovski, 2014; Harrison et al., 2015) observed in this class of genes across a wide variety of species. At the same time, and consistent with the molecular signatures observed, other work has suggested that sex‐biased genes represent resolved conflict, and therefore exhibit lower average levels of balancing selection than unbiased genes (Connallon & Knowles, 2005; Innocenti & Morrow, 2010; Mank, 2009; Wright et al., 2018). If broadly true, this suggests that conflict is prevalent in genes with similar expression patterns between the sexes, and is primarily resolved through regulatory decoupling of males and females into separate male and female genetic architectures. This conclusion is intuitively concordant with the fact that sex‐biased genes are primarily expressed in either males or females, and also suggests that sexual conflict is a short‐lived constraint, given the rapid turnover in sex‐biased gene expression across related species (Harrison et al., 2015; Zhang et al., 2007).

Importantly, recent theoretical work indicates that implausibly large selective pressures and mortality loads are required to generate the patterns of intersexual *F*_ST_ observed in the literature attributed to ongoing sexual antagonism (Kasimatis, Nelson, & Phillips, 2017; Kasimatis, Ralph, & Phillips, 2019). This calls into question the application of *F*_ST_‐based approaches for detecting sexual conflict arising from survival differences between the sexes. Consistent with this, a recent study found evidence that elevated intersexual *F*_ST_ for sex‐biased genes is actually the product not of sexual conflict, but of sex‐specific genetic architecture (Wright et al., 2018), where an allele only affects one sex or the other. Sex‐specific genetic architecture invokes relatively lower genetic loads, and there is increasing evidence that many loci exhibit profound sex differences in their phenotypic effects (Dapper & Wade, 2016; Gilks, Abbott, & Morrow, 2014; Karp et al., 2017). Similarly, recent analyses of large genomic data sets identified only a very small number of loci subject to antagonistic selection on survival (Czorlich et al., 2018; Mostafavi et al., 2017).

Furthermore, a major challenge in evolutionary biology is to explain the maintenance and variation in genetic diversity across many species. The existence of elevated genetic diversity relative to neutral expectations across species is puzzling, as directional selection and drift are both expected to erode variation. However, there is increasing evidence that intralocus sexual conflict, through balancing selection, can significantly increase genome‐wide patterns of variability (Chippindale, Gibson, & Rice, 2001; Delcourt, Blows, & Rundle, 2009; Foerster et al., 2007; Hawkes et al., 2016; Lonn et al., 2017; Mokkonen et al., 2011). Therefore, variation in sexual conflict across lineages, probably mediated by mating systems, could drive variation in genetic diversity across species and resolve this apparent paradox. However, the exact nature of the relationship between sexual conflict, mating system and genetic diversity remains unclear. Sexual conflict also has important implications for sexual selection, adaptation and evolvability. For instance, on the one hand, balancing selection would be expected to slow rates of sequence evolution arising from directional selection. However, balancing selection can also facilitate rapid adaptation from standing variation by maintaining multiple alleles within the population at high allele frequencies (Charlesworth, 2006; Hartl & Clark, 2006).

To assess the degree to which sex‐biased genes exhibit signatures of unresolved conflict and the potential for mating systems to exert balancing selection through sexual conflict on the genome, it is necessary to compare population genomic patterns of species and tissues with different levels of sexual dimorphism. We therefore estimated population genomic statistics for genes expressed in reproductive and somatic tissue across six avian species spanning the full range of mating systems and sexual selection in birds. Reproductive tissue has multiple sex‐specific functions and is phenotypically more sexually dimorphic, whereas the function of many somatic tissues is largely similar in males and females. By exploiting natural variation in the magnitude of sexual conflict across the body plan within individuals, as well as across mating systems between species, we were able to study the manifestation and resolution of sexual conflict, and subsequent genomic and phenotypic consequences. Our results reveal that the resolution of genomic sexual conflict is associated with the evolution of phenotypic sex differences. We demonstrate a clear link between variation in sexual conflict over reproduction and levels of genetic variation across phylogenetic space in a comparative framework.

## 2 MATERIALS AND METHODS

### 2.1 Tissue collection

**Figure 1:**
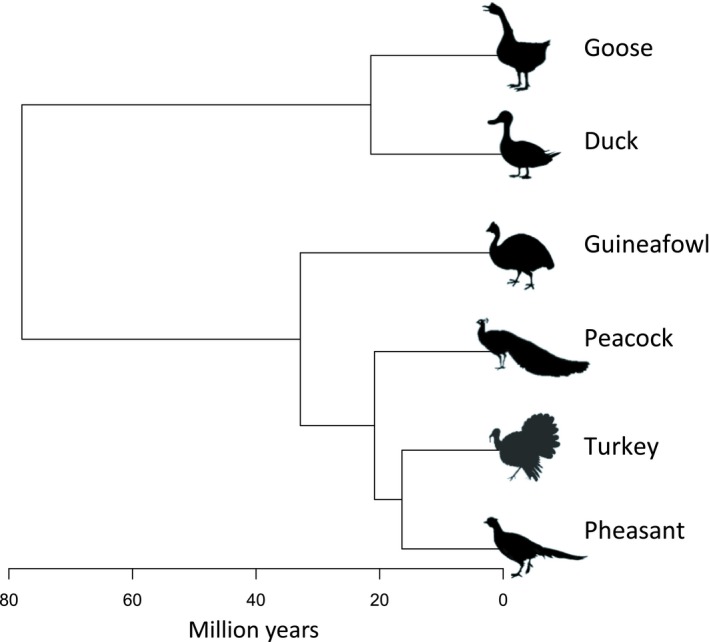
Phylogenetic relationships across the six avian species in this study. These species were chosen to reflect the full range of mating system and sexual dimorphism. The intensity of sexual conflict in each species was estimated using three proxies: sexual dichromatism score, sperm number and relative testes size

We previously extracted RNA from the left gonad and spleen of individuals with the RNeasy Kit (Qiagen), following the manufacturer's instructions, from the following captive avian populations: mallard (*Anas platyrynchos*), wild turkey (*Meleagris gallopavo*), common pheasant (*Phasianus colchicus*), helmeted guinea fowl (*Numida meleagris*), Indian peafowl (*Pavo cristatus*) and swan goose (*Anser cynoides*) (Harrison et al., 2015) (Figure 1). These captive populations are not maintained under sterile or biosafety conditions. Samples were collected during the first breeding season from five males and five females of each species, with the exception of the pheasant, where six male gonad and spleen samples were collected, and turkey where four male and two female spleens were collected.

These six species were deliberately chosen to reflect a full range of sexual dimorphism, ranging from monogamous and sexually monomorphic species such as the swan goose and guinea fowl, to polygynous and sexually dimorphic species such as the peafowl and wild turkey. We estimated the intensity of sexual conflict in each species using three proxies of sperm competition and male promiscuity: sexual dichromatism score, sperm number and relative testes size, obtained from Harrison et al., 2015.

### 2.2 Transcriptome assembly

Samples were sequenced on an Illumina HiSeq 2000 device with 100‐bp paired‐end reads and are available in the NCBI SRA (BioProject ID PRJNA271731). We assembled and filtered transcriptomes for each species using previously implemented approaches (Harrison et al., 2015). Briefly, we quality filtered RNA data using trimmomatic version 0.36 (Bolger, Lohse, & Usadel, 2014) to filter reads containing adaptor sequences and trim reads if the sliding window average Phred score over four bases was < 15 or if the leading/trailing bases had a Phred score < 3. Reads were removed after filtering if either read pair was < 36 bases in length. We assembled a de novo transcriptome for each species using trinity version 2.4.0 (Grabherr et al., 2011) with default parameters. We then filtered each transcriptome to remove spurious and low‐confidence genes. First, we selected the “best isoform” per gene to avoid redundancy. We used the trinity script align_and_estimate_abundance.pl to map RNA‐seq reads to transcriptomes using BOWTIE 2 and to quantify expression for each sample using rsem. We suppressed unpaired and discordant alignments for paired reads. We then picked the most highly expressed isoform per gene to obtain a set of “best isoforms” for each species. RNA‐seq reads were remapped to the set of “best isoforms” in each species using the same approach as above to ensure consistency between expression and sequence data. Second, we filtered the transcriptome to remove lowly expressed genes. Specifically, we removed genes with expression < 2 FPKM (fragments per kilobase of transcript per million mapped reads) in half or more of the individuals in either tissue. We assessed the completeness of our transcriptome assembly using eukaryota_odb9 busco version 3.0.2 (Waterhouse et al., 2018) (Table S1).

### 2.3 Identification of orthologues

We used blast (Altschul, Gish, Miller, Myers, & Lipman, 1990) to identify orthologous genes across the six species. First, we identified pairwise reciprocal orthologues between the chicken reference genome (Gallus_gallus‐5.0) and the wild turkey, common pheasant, helmeted guinea fowl and Indian peafowl, and between the duck reference genome (BGI_duck_1.0) and mallard and swan goose (Zerbino et al., 2018). We downloaded cDNA sequences from Ensembl (Zerbino et al., 2018) and selected the longest transcript per gene. We ran reciprocal blastn with an e‐value cut‐off of 1 × 10^−10^ and selected the best hit reciprocal orthologue using a minimum percentage identity of 30% and the highest bitscore following previous approaches (Harrison et al., 2015; Wright et al., 2018). If two hits shared the same highest bitscore, then the hit with the highest percentage identity was chosen. If both hits had the same highest bitscore and percentage identity, the gene was discarded.

For the wild turkey, common pheasant, helmeted guinea fowl and Indian peafowl, we assigned chromosomal location and gene position from the pairwise reciprocal orthologue in the chicken reference genome. Chromosomal positional information is not available in the duck reference genome and so we used a synteny‐based approach to obtain chromosomal location using mscanx (Wang et al., 2012). Briefly, we downloaded chicken and duck protein sequences from Ensembl, selected the longest protein per gene in each species, and then conducted a reciprocal blastp with an e‐value cut‐off of 1 × 10^−10^. We restricted the number of blastp hits for each gene to the top five, generated gff files, and concatenated the duck and chicken results as recommended by mscanx. We then identified syntenic regions between the duck and chicken reference genome using mscanx run with default parameters. For the mallard and swan goose, we assigned chromosomal location and gene position from the syntenic information available for the pairwise reciprocal orthologue in the duck reference genome. For all species, we split genes into autosomal or Z‐linked based on location in the chicken reference genome (Table S1) as evolutionary forces including sexual conflict act differently across these genomic regions (Rice, 1984; Wright & Mank, 2013).

Second, we identified reciprocal orthologues using the same approach across all species using the chicken and duck reference genomes to assign chromosomal location. This resulted in 1,457 autosomal reciprocal orthologues, which we used to contrast population genetic statistics across species. Finally, potential immune loci were identified from Gene Ontology terms in Biomart in the chicken and duck reference genomes (Zerbino et al., 2018). Specifically, we removed all loci with the terms “immune” or “MHC” in their Gene Ontology annotations from subsequent analyses. This was to reduce any potential confounding effects as heterozygote advantage in immunity can produce patterns of balancing selection independent of sexual conflict (Ghosh, Andersen, Shapiro, Gerke, & Kruglyak, 2012; Hedrick, 2011; Stahl, Dwyer, Mauricio, Kreitman, & Bergelson, 1999).

### 2.4 Gene expression analyses

Read counts for autosomal and Z‐linked genes were extracted for all gonad and spleen samples and normalized using TMM in edger (Robinson, McCarthy, & Smyth, 2010). We identified gonad‐biased, spleen‐biased and non‐tissue‐biased genes using a standard log_2_ fold change value of 2 (Wright et al., 2018) in each species (Tables S2 and S3). The gonad is transcriptionally more sexually dimorphic than the spleen and so we identified tissue‐biased genes in each sex separately instead of combining all samples to avoid biasing our analyses against highly sex‐biased or sex‐limited genes. We report results from tissue‐biased genes identified in males in the main text but results based on tissue‐biased genes identified from female expression data are fully detailed in the Supporting Information. The results are qualitatively identical unless otherwise indicated. Sex‐biased genes were identified in each set of tissue‐biased genes using a log_2_ fold change value of 1. We identified tissue‐biased genes on the Z chromosome separately due to the unique expression profile of the avian Z chromosome arising from incomplete dosage compensation (Itoh et al., 2007; Mank & Ellegren, 2008; Wright, Moghadam, & Mank, 2012).

### 2.5 Filtering data for population genomic analyses

Population genomic analyses were conducted on BAM files generated by mapping RNA‐seq data to the set of “best isoforms” in each species with rsem. For each individual, we merged the spleen and gonad BAM files using samtools (Li et al., 2009). The exception was the turkey, where the spleen and gonad were not sequenced for all individuals so we used only gonad data for subsequent analyses.

We used angsd (Korneliussen, Albrechtsen, & Nielsen, 2014) to estimate population genetic summary statistics, following our previous approach (Wright et al., 2018) as angsd implements methods to account for sequencing uncertainty and is appropriate for uneven sequencing depth associated with transcriptome data. We filtered BAM files to discard reads if they did not uniquely map, had a flag ≥ 256, had a mate that was not mapped or had a mapping quality below 20. Bases were filtered if base quality fell below 13 or there was data in fewer than half the individuals. Mapping quality scores were adjusted for excessive mismatches and quality scores were adjusted around indels to rule out false single nucleotide polymorphisms (SNPs).

We identified and removed related individuals (four peacock, two wild turkey and two swan goose individuals) from our analyses using ngsrelate (Korneliussen & Moltke, 2015) to avoid violating Hardy–Weinberg assumptions, and calculated inbreeding coefficients using an EM algorithm with the ngsf package in ngstools (Fumagalli, Vieira, Linderoth, & Nielsen, 2014) (full details in Methods S1). For all species, inbreeding coefficients were < 0.03 with the exception of the peacock where we identified two inbred individuals. We incorporated inbreeding coefficients for the peacock in subsequent analyses.

### 2.6 Calculating Tajima's *D*

angsd was used for each species to calculate sample allele frequency likelihoods at each site from genotype likelihoods calculated with the samtools model. We calculated allele frequency likelihoods separately for the Z chromosome and the autosomes as they are subject to different evolutionary pressures and differ in ploidy. The Z chromosome is diploid in males yet haploid in females, and therefore we used only male samples to estimate allele frequency to avoid violating Hardy–Weinberg assumptions. Next, we estimated the overall unfolded site frequency spectrum (SFS) for each species (Nielsen, Korneliussen, Albrechtsen, Li, & Wang, 2012) (Figure S1). Specifically, at each site we randomly sampled an allele frequency according to its likelihood, as calculated by ansgd. Finally, we computed genetic diversity indices, including allele frequency posterior probability and Tajima's *D* using the SFS as prior information with angsd thetaStat (Korneliussen et al., 2014).

For each species, we calculated a relative measure of Tajima's *D* for spleen‐biased and gonad‐biased genes. Specifically, we quantified median *D* relative to non‐tissue‐biased genes, our neutral estimate of *D* for each species. Calculating a relative measure of Tajima's *D* makes it possible to circumvent problems arising from demographic changes in population size that would otherwise bias comparative analyses of population genetic statistics across species.

### 2.7 Calculating intersexual* F*_ST_

Intersexual *F*_ST_ was calculated using the same procedure and filtering criteria as Tajima's *D*, except that RNA‐seq data were instead filtered to remove bases where we had data in fewer than half the individuals in males and females separately. This ensures we do not exclude sex‐limited genes from the analysis. Hudson's *F*_ST_, which is less sensitive to small sample sizes (Bhatia, Patterson, Sankararaman, & Price, 2013), was estimated as implemented in angsd (Korneliussen et al., 2014). Estimates across loci were obtained using weighted averages (see Fumagalli et al 2014, Equations 4 and 12), where per‐gene *F*_ST_ is the ratio between the sum of the between‐populations variance across loci and the sum of the total variance across loci. Given the Z chromosome is haploid in females, we do not have the power to analyse patterns of *F*_ST_ across the Z chromosome in this study.

## 3 RESULTS

### 3.1 Lower levels of ongoing sexual conflict in reproductive versus somatic tissue

Reproductive tissue, such as the gonad, has many sex‐specific functions whereas the function of somatic tissue, such as the spleen, is more aligned between male and female fitness. To test whether phenotypic sexual dimorphism is associated with resolved sexual conflict at the genomic level, we contrasted population genomic statistics between genes expressed in the gonad versus the spleen.

**Figure 2:**
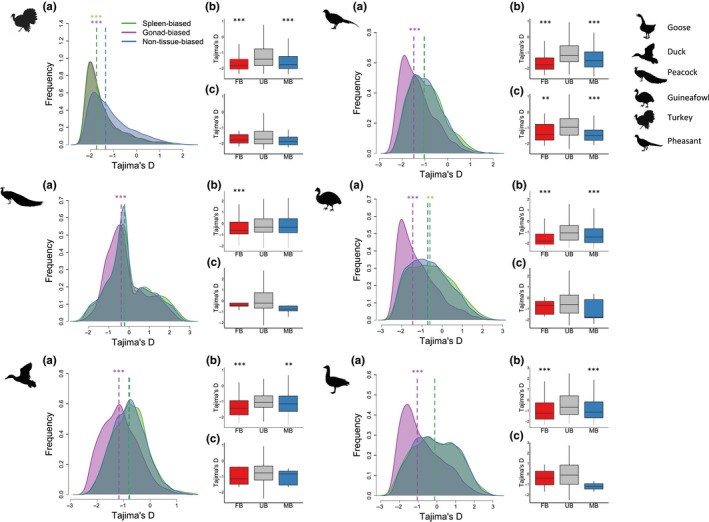
Patterns of Tajima's *D* for tissue‐biased and sex‐biased genes across species. (a) The distribution of *D* for autosomal genes for spleen‐biased, gonad‐biased and non‐tissue‐biased genes. Dotted lines show median *D* for each set of genes and asterisks denote a significant difference relative to non‐tissue‐biased genes (Wilcoxon test, *p < 0.05, **p < 0.01, **p < 0.001). Tissue‐biased genes were identified from male expression data. (b, c) The relationship between *D* and expression for genes with gonad‐biased expression (b) or spleen‐biased expression (c). Asterisks denote a significant difference relative to unbiased genes (Wilcoxon test, *p < 0.05, **p < 0.01, **p < 0.001). FB, UB, MB refer to female‐biased, unbiased and male‐biased genes, respectively [Colour figure can be viewed at wileyonlinelibrary.com]

As heterozygote advantage in immunity can produce patterns of balancing selection independent of sexual conflict (Ghosh et al., 2012; Hedrick, 2011; Stahl et al., 1999), we removed all loci with potential immune function from downstream analyses. We found that median Tajima's *D* is significantly lower for gonad‐biased genes relative to genes expressed in both tissues in all species across the autosomes (Figure 2a and Figure S2A). This result is consistent with lower levels of ongoing sexual antagonism in the gonad. In contrast, we found no significant difference in Tajima's *D* between spleen‐biased genes and loci expressed in both tissues in the majority of species. We observed consistent patterns on the Z chromosome (Figure S5), although our power to detect statistically significant differences is reduced due to limited numbers of tissue‐biased Z‐linked genes (Table S1).

The proportion of sex‐biased genes varies across the spleen and gonad (Harrison et al., 2015) and sex‐biased genes are subject to different selective pressures (Ellegren & Parsch, 2007; Harrison et al., 2015) as well as distinct patterns of balancing selection relative to unbiased genes (Cheng & Kirkpatrick, 2016; Dutoit et al., 2018; Wright et al., 2018). To ensure that differences in the number of sex‐biased genes between the two tissues are not responsible for the lower Tajima's *D* we observe in gonad‐biased genes, we repeated the analyses using Tajima's *D* calculated only from unbiased genes in each tissue. We found a consistent pattern across the majority of species, where Tajima's *D* is significantly lower in gonad‐biased but not spleen‐biased genes relative to loci expressed similarly in both tissues (Figure S3). However, these species differ in mating system, which could explain the variation in the strength of balancing selection we observe across species, addressed in more detail below.

It is important to note that multiple factors can influence population genetic statistics for any particular locus. Therefore, we tested whether our results could also be attributed to the effect of covariates that might vary across tissue‐biased genes. We incorporated measures of gene length, average expression level, GC content and Watterson's theta into a multiple regression (TD ~ Tissue bias + log(tW) + log(Gene length) + log(GC) + log(Gene expression level)). Tissue‐bias remains a significant factor in explaining variation in Tajima's *D* once accounting for these covariates (Table S11). However, the effect size in some species is relatively small, indicating that the pattern we detect is subtle and influenced by multiple factors.

### 3.2 Limited power of intersexual *F*_ST_ to detect sexual conflict arising over survival

We tested the power of intersexual *F*_ST_ to detect sexual conflict arising over survival through contrasts between the spleen and gonad. Given its role in the lymphatic system and in filtering blood components, we might expect the spleen to be subject to viability selection more so than the gonad, whose role is primarily reproductive. We removed sex‐biased genes from this analysis to avoid biasing the results, as the abundance of sex‐biased expression differs between reproductive and somatic tissue and previously we have shown that intersexual *F*_ST_ is often elevated for sex‐biased genes (Cheng & Kirkpatrick, 2016; Dutoit et al., 2018; Wright et al., 2018).

We contrasted intersexual *F*_ST_ for gonad‐ and spleen‐biased genes using three approaches. First, we found no significant difference in median *F*_ST_ for unbiased genes expressed primarily in the gonad relative to those expressed broadly across both the gonad and the spleen (Table S4). We observed the same pattern in the spleen, with the exception of the goose and turkey where *F*_ST_ was elevated marginally. Second, there was no significant difference in the number of unbiased genes with elevated intersexual *F*_ST_ that were expressed primarily in the gonad compared to those expressed in both tissues (Table 1). We observe the same result in the spleen, with the exception of the turkey. However, all of these differences become nonsignificant when we analyse tissue‐biased genes identified from female expression data (Table S5 and Table S6). Last, we found no significant effect of tissue bias on *F*_ST_ after accounting for gene length, average expression level, GC content and Watterson's theta in a multiple regression (TD ~ Tissue bias + log(tW) + log(Gene length) + log(GC) + log (Gene expression level)) (Table S11).

**Table 1:** Observed and expected number of genes with intersexual *F*_ST_ > 0 across tissue‐biased genes

Species	Gonad‐biased			Spleen‐biased		
	E	O	*p*‐value	E	O	*p*‐value
Mallard	116	118	0.875	112	111	0.956
Swan goose	56	65	0.248	56	70	0.056
Wild turkey	166	160	0.644	204	236	**0.026** a
Common pheasant	165	163	0.520	187	174	0.532
Guinea fowl	112	124	0.269	151	142	0.461
Indian peafowl	200	209	0.520	217	208	0.532

Note

Only unbiased genes were used in this analysis. Tissue‐biased genes were identified from male expression data. Only autosomal genes are included in the analyses. The expected number of genes with intersexual *F*_ST_ > 0 was calculated from observations of *F*_ST_ in non‐tissue‐specific genes. *p*‐values were calculated using chi‐squared tests.

a *p*‐values in bold are significant (*p* < 0.05)

Intriguingly, despite the limited potential role of the gonad in survival, elevated intersexual *F*_ST_ has been previously detected in gonad‐expressed genes in flycatchers (Dutoit et al., 2018). Consistent with this, we find a weak relationship between intersexual *F*_ST_ and sex‐biased gene expression in the gonad, where *F*_ST_ is significantly elevated in sex‐biased genes in some species (Figure S7, Table S12). However, note that our power to quantify intersexual *F*_ST_ is limited by our sample size. Whilst our results are consistent with flycatchers, the associated effect sizes are weak (sex‐bias and *F*_ST_ for gonad‐biased genes *r* ^2^ = 0.000–0.042, spleen‐biased genes *r* ^2^ = 0.000–0.008). Most importantly, our results are consistent with theoretical work suggesting that intersexual divergence in allele frequency may not always be a reliable indicator of ongoing sexual conflict over viability (Kasimatis et al., 2017, 2019), particularly in studies with low numbers of samples.

### 3.3 Regulatory evolution is associated with resolved conflict over long evolutionary time frames

We contrasted population genomic statistics across sex‐biased and unbiased genes to test the role of regulatory variation in sexual conflict resolution. We found that autosomal sex‐biased genes expressed in the gonad have significantly lower Tajima's *D* than unbiased genes across all six species, consistent with largely resolved sexual conflict (Figure 2 and Figure S2). However, male‐ and female‐biased genes also have significantly elevated intersexual *F*_ST_ in many species (Figure S7), even after accounting for potential covariates (Table S12). These results are consistent with a potential role of regulatory evolution in conflict resolution via the evolution of sex‐specific architecture (Wright et al., 2018). We observed a similar pattern across spleen‐biased genes (Figure 2 and Figure S2), although the differences are nonsignificant, probably because of reduced power due to limited numbers of sex‐biased genes in somatic tissue.

Employing discrete thresholds to identify sex‐biased genes has been shown to have a major effect on the number of genes identified (Ingleby, Flis, & Morrow, 2015). We therefore next investigated the relationship between Tajima's *D* and sex‐bias using a polynomial approach (Cheng & Kirkpatrick, 2016). These results confirmed our finding that sex‐biased genes have lower Tajima's *D* (Tables S7, S8, S9 and S10). It is important to note that the variance in Tajima's *D* that is accounted for by these associations is extremely low (sex‐bias and *D* for gonad‐biased genes *r* ^2^ = 0.007–0.147, spleen‐biased genes *r* ^2^ = 0.000–0.018), similar to findings of previous somatic studies in fish (Wright et al., 2018), probably resulting, at least in part, from the inherent noise in Tajima's *D* estimates.

To quantify the pervasiveness of sexual conflict and extent to which balancing selection shapes patterns of genetic diversity across related species, we identified reciprocal orthologues across the six species, which last shared a common ancestor 90 million years ago. Across reciprocal orthologues on the autosomes, we identified genes with elevated Tajima's *D* in all species: specifically, where Tajima's *D* was in the top 10% quantile in each species separately. The average range of Tajima's *D* values for this highest 10% class across species was 1.41–3.26. Using ancestral reconstructions of gene expression levels (Harrison et al., 2015) (Methods S1), we identified gonadal genes that were ancestrally and universally either sex‐biased or unbiased across all six species. We found that gonadal genes that were ancestrally sex‐biased across the clade were significantly less likely to show elevated Tajima's *D* across all six species than expected from random permutations (245 genes, χ^2^ *p* < 0.001, 1,000 permutations). In contrast, universally unbiased genes were significantly enriched in genes with elevated Tajima's *D* across all species (141 genes, χ^2^ *p* < 0.001, 1,000 permutations). Our results are robust across multiple quantile thresholds used to define elevated Tajima's *D* (Results S1). This indicates that sexual conflict can shape patterns of genetic diversity in certain sets of sex‐biased genes across evolutionary time frames.

### 3.4 Conflict over reproductive potential is greatest in sexually dimorphic species

To investigate the relationship between sexual conflict and levels of genetic diversity across the genome, we conducted a phylogenetically controlled comparative analysis of Tajima's *D* across species that vary in mating system and sexual dimorphism. Specifically, we used phylogenetic generalized least squares (PGLS) from the R package caper (Orme et al., 2013) to test the relationship between Tajima's *D* and measures of sexual dimorphism, while accounting for the observed level of phylogenetic signal in the data. For each species, we quantified median Tajima's *D* for spleen‐ and gonad‐biased genes relative to non‐tissue‐biased genes. Tajima's *D* cannot be compared directly across species or populations, as demographic history has a major influence on genetic diversity, and therefore on Tajima's *D* estimation. Calculating a relative measure of Tajima's *D* makes it possible to circumvent problems arising from demographic changes in population size. There are a number of phenotypic indices of sexual conflict, including degree of sexual dichromatism, sperm number and residual testes weight, that are widely used indicators of post‐copulatory sexual selection and therefore a measure of variance in male mating success in birds (Birkhead & Moller, 1998; Moller, 1991; Pitcher, Dunn, & Whittingham, 2005). We recovered a significant and positive relationship between relative Tajima's *D* in the gonad and sexual dichromatism (*r* ^2^ = 0.890, *p* = 0.003) after correcting for phylogeny, and marginally nonsignificant positive associations with both sperm number (*r* ^2^ = 0.491, *p* = 0.073) and residual testes weight (*r* ^2^ = 0.298, *p* = 0.152).

**Figure 3:**
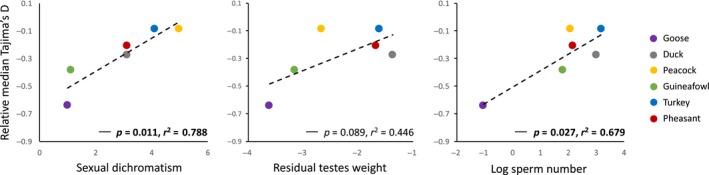
Phylogenetically controlled regression between proxies of sperm competition and Tajima's *D* in the gonad. Relative *D* is shown for autosomal genes with unbiased expression between males and females in the gonad. Relative *D* is calculated as the difference between median *D* for tissue‐biased genes compared to non‐tissue‐biased genes. Tissue‐biased genes were identified from male expression data. We tested the relationship between Tajima's *D* and measures of sexual dimorphism, while accounting for the observed level of phylogenetic signal in the data [Colour figure can be viewed at wileyonlinelibrary.com]

The proportion of sex‐biased genes varies with mating system across these species (Harrison et al., 2015), which together with the fact that sex‐biased genes have distinct patterns of Tajima's *D* (Cheng & Kirkpatrick, 2016; Dutoit et al., 2018; Wright et al., 2018) and are subject to different selective pressures relative to unbiased genes (Ellegren & Parsch, 2007; Harrison et al., 2015), may confound the pattern we observe. We therefore repeated the analyses using relative median Tajima's *D* calculated using only unbiased genes in each tissue. In doing so, we found that relative Tajima's *D* in the gonad becomes significantly and positively correlated with sexual dichromatism (*r* ^2^ = 0.788, *p* = 0.011), and sperm number (*r* ^2^ = 0.679, *p* = 0.027) after correcting for phylogenetic relationships (Figure 3), and marginally nonsignificantly associated with residual testes weight (*r* ^2^ = 0.446, *p* = 0.089). In contrast, there was no significant association with Tajima's *D* in the spleen and measures of sexual dimorphism (Figure S4).

Interestingly, we found no significant relationship between Tajima's *D* and phenotypic sexual conflict for Z‐linked genes in either tissue (Figure S6). Given there are fewer genes on the Z chromosome relative to the autosomes, this pattern might simply be a consequence of smaller sample sizes and therefore greater uncertainty around the median. To assess the role of gene number in our population genetic parameter estimates, we subsampled tissue‐biased genes on the autosomes to the equivalent number of the Z‐linked genes in each species 1,000 times. The Pearson's correlation coefficients for the relationship between Tajima's *D* and sexual dichromatism, testes weight and sperm number for gonad‐biased Z‐linked genes are smaller relative to the subsampled data set (*p* = 0.027, *p* = 0.048, *p* = 0.168). The slope of the regression is also smaller than the subsampled data (*p* = 0.024, *p* = 0.058, *p* = 0.121). This indicates that our failure to observe a significant relationship between Tajima's *D* and sexual conflict on the Z chromosome is not a consequence of reduced gene numbers relative to the autosomes.

## 4 DISCUSSION

The manifestation, resolution and consequences of intralocus sexual conflict have been the subject to considerable recent debate. To address this, we exploited natural variation in the magnitude of sexual conflict across the body plan within individuals, and across mating systems between species, in a clade of birds that diverged 90 million years ago.

The role of regulatory variation between males and females in the resolution of sexual conflict has received substantial attention in recent literature, with population genomic studies suggesting that sex‐biased genes are subject to ongoing sexual antagonism (Cheng & Kirkpatrick, 2016; Dutoit et al., 2018) and others indicating that they represent resolved conflict (Innocenti & Morrow, 2010; Wright et al., 2018). Sex‐biased genes in the guppy tail, particularly male‐biased genes, resolve conflict arising over reproduction through the evolution of separate sex‐specific genetic architectures (Wright et al., 2018). However, as this tissue is heavily implicated in female mate choice and therefore primarily affects male reproductive fitness, it is possible that the relative importance of male versus female expression is unusual in this tissue and that sex‐biased genes play equal roles in most species. Contrary to this, Dutoit et al. (2018) suggest that ongoing sexual antagonism is more prevalent in male‐ than female‐biased genes in the gonad, potentially hinting at an important role for female‐biased expression in conflict resolution. However, without a direct comparison between sex‐biased and unbiased genes, the relationship remains unclear. Finally, both male‐ and female‐biased genes in humans show elevated *F*_ST_ measures (Cheng & Kirkpatrick, 2016), although it is not clear how much of this signal is due to somatic versus gonadal expression, or whether this was associated with elevated Tajima's *D*.

Here, we find that balancing selection is weaker in sex‐biased genes relative to unbiased genes, consistent with an important role for sex‐biased expression in the resolution of sexual conflict. Lower Tajima's *D* in sex‐biased genes is consistent with the rapid rates of evolution in this class of genes observed across many species (Ellegren & Parsch, 2007; Mank, 2017; Parsch & Ellegren, 2013; Rowe et al., 2018), either through positive selection (Meiklejohn et al., 2003; Pröschel et al., 2006; Zhang et al., 2007) or relaxed purifying selection (Dapper & Wade, 2016; Dutoit et al., 2018; Gershoni & Pietrokovski, 2014; Harrison et al., 2015). Balancing selection, which slows the fixation of alleles, is inconsistent with accelerated rates of sequence evolution observed for sex‐biased genes (Harrison et al., 2015; Wright & Mank, 2013). In contrast, resolved conflict, which results in sex‐specific selection and separate male and female genetic architectures suggested by our data, is expected to lead to the higher levels of standing diversity and faster rates of evolution observed across sex‐biased genes in a broad array of taxa (Dapper & Wade, 2016).

Whereas identifying the mechanisms responsible for the resolution of genomic sexual conflict has received considerable attention, the consequences for phenotypic evolution have been comparatively understudied. This is in part due to the difficulties in identifying specific loci subject to sexual conflict and establishing their phenotypic effects from genome scans alone. Our study adds considerably to this goal by using different levels of dimorphism within the body plan and across related species to determine the relationship between population genetic and phenotypic measures of sexual conflict.

Relative to the spleen, the gonad is more phenotypically sexually dimorphic, has higher levels of sex‐biased gene expression, and has evolved many sex‐specific functions. If sexual dimorphism represents resolved sexual conflict, we might expect gonad‐biased genes to have lower levels of balancing selection than spleen‐biased genes and loci expressed similarly in both tissues. Consistent with this prediction, we find reduced balancing selection in the gonad, indicative of lower levels of ongoing sexual conflict. This supports the theory that resolved sexual conflict facilitates the evolution of phenotypic sex differences. It is plausible that the large numbers of sex‐biased genes in the gonad relative to somatic tissue act to resolve conflict through regulatory decoupling of male and female expression and the evolution of sex‐specific architecture.

While we found that intralocus sexual conflict is resolved in the gonad, we found a significant and positive correlation between the magnitude of sexual conflict, arising from differences in mating system, and balancing selection in the gonad but not the spleen. Whilst this may appear initially contradictory, this relationship is in fact consistent with an ephemeral nature of sexual antagonism and rapid turnover of sexual conflict loci. This is in line with previous work showing that sex‐biased genes exhibit rapid rates of evolution and turnover (Harrison et al., 2015; Zhang et al., 2007). Our results suggest that unbiased genes are the locus of ongoing sexual conflict due to mating system, and that increasing levels of sexual conflict over reproduction result in elevated levels of genetic diversity across a greater proportion of genes. In contrast, relative Tajima's *D* in spleen‐biased genes is not associated with any phenotypic measure of sexual conflict, suggesting that sexual conflict over reproduction has the greatest potential to contribute significantly to variation in the maintenance of genetic diversity across species. This has important consequences for understanding the relationship between sexual conflict and adaptation, where higher levels of conflict promote genetic diversity and provide genetic fuel for adaptive opportunities (Candolin & Heuschele, 2008; Chenoweth, Appleton, Allen, & Rundle, 2015; Jacomb, Marsh, & Holman, 2016; Lumley et al., 2015).

In contrast, we observed no significant relationship between mating system and balancing selection on the Z chromosome. Previously, we showed that the adaptive potential of the Z chromosome is compromised by increasing sexual selection, which decreases the relative effective population size of the Z chromosome compared to autosomes (Wright et al., 2015), leading to increased levels of genetic drift. This means that Z‐linked genes in sexually dimorphic species are subject to higher levels of genetic drift (Wright & Mank, 2013). Our results indicate that the potential for sexual conflict to shape patterns of genetic diversity on the Z chromosome might be counteracted by the depleting forces of genetic drift, and that sexual conflict may not play a disproportionally greater role in Z chromosome evolution compared to the rest of the genome.

Negative Tajima's *D* can be interpreted in the context of positive selection, where selective sweeps can result in lower estimates. A greater frequency of selective sweeps in sex‐biased genes could therefore explain our finding that Tajima's *D* is lower in the gonad than in the spleen. Furthermore, the positive correlation between Tajima's *D* and sexual dimorphism we observe in the gonad could also be due to more intense positive selection in species with less sexual dimorphism. However, elevated positive selection is unlikely to explain our results, as previous research on the same data set found no significant evidence for positive selection acting on sex‐biased genes in the gonad, or any evidence for variation in the magnitude of positive selection across species based on mating system (Harrison et al., 2015). Therefore, we conclude that lower Tajima's *D* is indicative of lower levels of balancing selection and resolved intralocus conflict, probably mediated by the evolution of sex‐biased gene expression.

Population genomic measures of intersexual *F*_ST_ and Tajima's *D* can be influenced by a number of demographic events, not just sexual conflict, including sex‐biased migration, sex‐biased predation and changes in population size (Hartl & Clark, 2006). By conducting comparisons of population genomic statistics within each species, instead of directly comparing across species, we controlled for the effect of population contractions or expansions, and our use of captive populations further minimizes the effects of sex‐biased migration or predation. Furthermore, samples were taken from all individuals during their first breeding season, effectively controlling for age differences that can confound measures of intersexual *F*_ST_ or lead to high levels of regulatory variation. However, we note that due to statistical noise, probably due to low sample sizes, we could not reliably identify specific loci subject to sexual conflict, and instead compare large groups of genes to determine broad trends across tissues and species. Our analyses of intersexual *F*_ST_ are particularly limited by sample size and therefore we urge caution when interpreting these in the light of sexual conflict. However, while we do find loci with elevated intersexual *F*_ST_, which has previously been interpreted as evidence for ongoing sexual conflict (Cheng & Kirkpatrick, 2016; Dutoit et al., 2018; Lucotte et al., 2016), the number of loci with elevated *F*_ST_ do not appear to differ between the gonad and spleen, despite the obvious differences in function and role in survival between the two tissues.

Interestingly, our failure to detect differences in conflict over viability between the tissues is consistent with recent theoretical work (Kasimatis et al., 2017) suggesting that the magnitude of sexual conflict, and associated mortality load, required to generate patterns of intersexual *F*_ST_ across large numbers of loci is implausibly high. This suggests that they may be a result of alternative demographic processes or statistical noise arising from low sample sizes, instead of ongoing sexual conflict. Instead, our previous work indicates that divergence in allele frequencies between males and females in somatic tissue could instead be indicative of the evolution of sex‐specific architectures, which would invoke weaker genetic loads.

In conclusion, our findings suggest that mating system can significantly increase standing diversity across the genome via sexual conflict. More importantly, our results suggest that sexual conflict is short‐lived, and is resolved via the decoupling of male and female gene expression patterns. Our results are consistent both across a gradient of sexual dimorphism within the body plan and across species, and have important implications regarding the role of sexual selection in adaptive potential (Candolin & Heuschele, 2008; Chenoweth et al., 2015; Jacomb et al., 2016; Lumley et al., 2015), the persistence of sexual conflict over evolutionary timescales, and the role of dimorphism in facilitating sex‐specific fitness optima.

## Data Availability

RNA‐seq data are publicly available in the NCBI SRA (BioProject ID PRJNA271731). Transcriptome assemblies are available via Dryad (https://doi.org/10.5061/dryad.1v2d850). Statistics for autosomal genes in each species are available in Supporting Information data files.
