# Supplementary material for: Phenotypic sexual dimorphism is associated with genomic signatures of resolved sexual conflict
Source: Mol Ecol. 2019 Jun 5;28(11):2860–71. doi: 10.1111/mec.15115 (PMC6618015; doi:10.1111/mec.15115)
Supplement: Supplementary file 1 [file MEC-28-2860-s001.zip › mec15115-sup-0001-Supinfo.pdf]

MOLECULAR ECOLOGY

Supplemental Information for:

Phenotypic sexual dimorphism is associated with genomic signatures of resolved sexual conflict

Alison E. Wright[1]*, Thea F. Rogers[1], Matteo Fumagalli[2], Christopher R. Cooney[1] & Judith E. Mank[3,4,5]

1 Department of Animal and Plant Sciences, University of Sheffield, United Kingdom
2 Department of Life Sciences, Silwood Park Campus, Imperial College London, United Kingdom
3 Department of Genetics, Evolution and Environment, University College London, United Kingdom
4 Department of Organismal Biology, Uppsala University, Sweden
5 Department of Zoology, University of British Columbia, Canada
* Corresponding author: a.e.wright@sheffield.ac.uk

Table of Contents:

Supplementary Data — Page 2
Supplementary Materials & Methods — Page 2
Supplementary Results — Page 4
Figure S1-7 — Page 5-11
Table S1-12 — Page 12-20
References — Page 21

MOLECULAR ECOLOGY

SUPPLEMENTARY DATA

Statistics for autosomal genes in each species in SI data files. Gene ID, expression (in female gonad, male gonad, female spleen, male spleen), Tajima's D, Pi, Watterson's theta, Intersexual F_{ST}, gene length, GC content. Population genomic statistics were calculated with ANGSD.

SUPPLEMENTARY MATERIALS & METHODS

Calculating relatedness and inbreeding coefficients

We identified and removed related individuals from our analyses using ngsRelate (Korneliussen and Moltke 2015) to avoid violating Hardy Weinberg assumptions. First, we estimated allele frequencies with ANGSD using the SAMtools genotype likelihood model and inferred the major and minor allele from the genotype likelihood. Sites with SNP pvalue > 1e-6 or with MAF < 0.05 were removed from the analysis. We extracted allele frequencies and calculated relatedness estimates for all pairs of individuals in each species using ngsRelate. We excluded four peacock, two wild turkey and two swan goose individuals from subsequent analyses.

We used ANGSD to estimate allele frequencies using the SAMtools genotype likelihood model and assuming the reference base being one of the two possible alleles. Triallelic sites and sites with SNP pvalue > 1e-6 were removed. We estimated pairwise linkage disequilibrium from unphased expected genotypes calculated from genotype likelihoods using ngsLD (Fox, et al. 2019). We then pruned linked sites using a threshold on r^2 of 0.3 and retrieved unlinked sites for further analysis.

Inbreeding coefficients were calculated using an EM algorithm with the ngsF package in ngsTools (Fumagalli, et al. 2014). The maximum root-mean-square deviation between iterations to assume convergence was 0.001. For all species, inbreeding coefficients were <0.03 with the exception of the peacock where we identified two inbred individuals.

Model selection for the relationship between sex-bias, Tajima's D, and intersexual F_{ST}

We followed our previously implemented approach (Wright, et al. 2018) based on Cheng and Kirkpatrick (2016) to fit a parametric model to describe the relationship between gene expression, Tajima's D, and intersexual F_{ST}. We regressed Tajima's D or intersexual F_{ST} and sex-biased expression using polynomials. The optimal polynomial degree was determined using the Akaike Information Criterion (AIC) (Burnham and Anderson 2003) in R (Team 2016) and likelihood ratio tests to assess significance of each model using lrtest function in the lmtest package in R (Zeileis and Hothorn 2002). Models within 2 AIC units of the model with the lowest AIC (or p <0.05) were treated as one top model set, and the model with the fewest parameters was preferred.

Ancestral state reconstruction

Ancestral state reconstruction of sex-biased expression followed the approach of Harrison et al. (2015). To produce a phylogeny with time-calibrated branch lengths for the species in our dataset, we downloaded 100 trees containing our focal species from www.birdtree.org (Jetz, et al. 2012), based on the Hackett et al. (2008) backbone. We then used TreeAnnotator (Drummond, et al. 2012) to generate a maximum clade credibility (MCC) tree, using median node heights. We used this phylogeny and a Brownian motion-based maximum likelihood method implemented in the 'ace' function in the APE package (Paradis, et al. 2004) to infer ancestral values for the extent of sex-biased expression. We then used these reconstructed values to infer whether genes were (i) ancestrally and universally sex-biased (i.e. all nodes reconstructed as sex biased) or (ii) unbiased (i.e. all nodes reconstructed as unbiased) across the galloanserae phylogeny.

From these universally sex-biased or unbiased genes, we identified genes with elevated Tajima's D across all species; specifically, where Tajima's D was in the top 10% quantile in each species. We chose this quantile threshold to ensure we were studying genes with significantly elevated Tajima's D and to maximise the number of genes. However, we repeated the analysis using a range of quantile values (SI Results).

MOLECULAR ECOLOGY

SUPPLEMENTARY RESULTS

Regulatory evolution is associated with resolved conflict over long evolutionary timeframes.

In order to quantify the pervasiveness of sexual conflict and extent to which balancing selection shapes patterns of genetic diversity across related species, we identified reciprocal orthologs across the six species, which last shared a common ancestor 90 million years ago. Across reciprocal orthologs on the autosomes, we identified genes with elevated Tajima's D in all species. We used multiple quantiles to classify elevated Tajima's D and our results are robust to different cut-offs. Gonadal genes that were ancestrally male- or female-biased across the clade were less likely to show elevated Tajima's D across all six species than expected from random permutations (15% quantile: $p<0.001$ Observed=140 Expected=221, 10% quantile: $p<0.001$ Observed=90 Expected=148, 5% quantile: $p=0.004$ Observed=49 Expected=73, 1% quantile: $p=0.3392$ Observed=11 Expected=16, Chi-squared test, 1000 permutations). In contrast, universally unbiased genes were enriched in genes with elevated Tajima's D across all species (15% quantile: $p<0.001$ Observed=178 Expected=127, 10% quantile: $p<0.001$ Observed=117 Expected=85, 5% quantile: $p =0.163$ Observed=51 Expected=42, 1% quantile: $p=0.576$ Observed=10 Expected=8, Chi-squared test, 1000 permutations).

MOLECULAR ECOLOGY

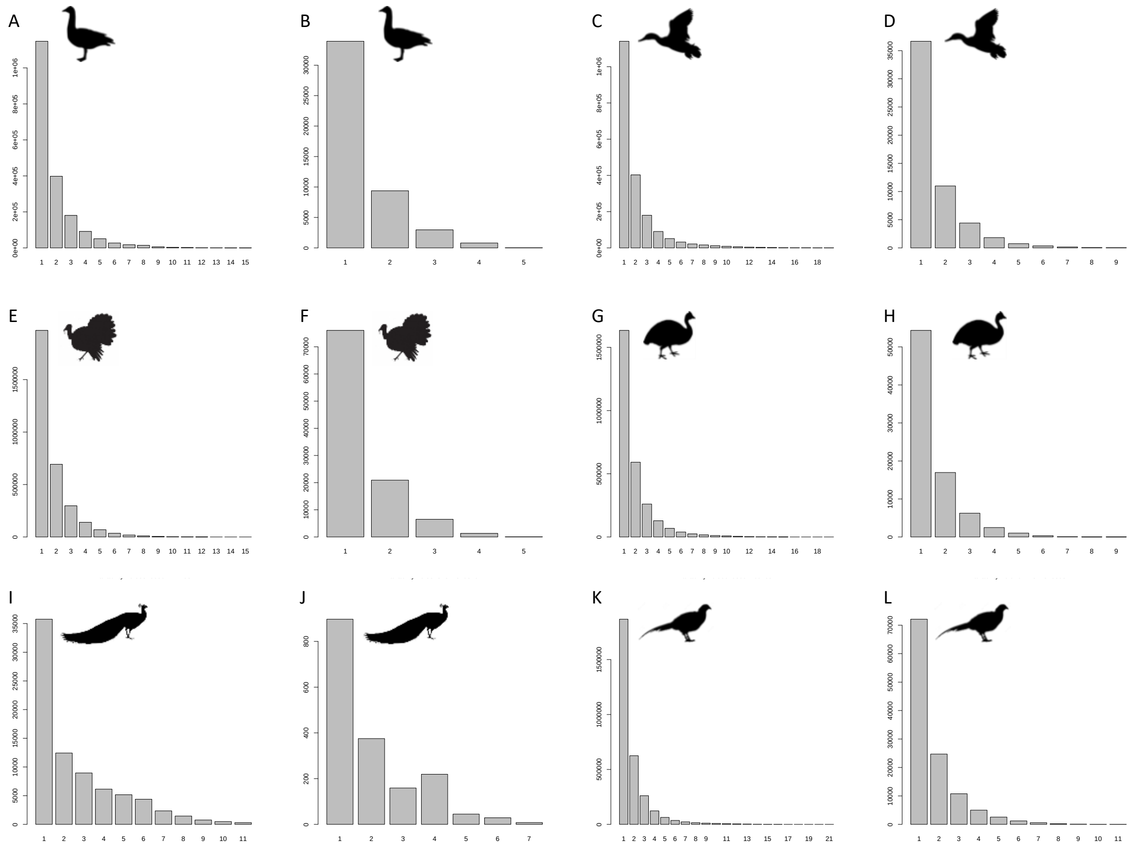

Figure S1. Site frequency spectrum for the Z chromosome and autosomes. Panels A, C, E, G, I, K show the autosomal SFS for the swan goose, mallard duck, wild turkey, helmeted guineafowl, peacock, common pheasant. Panels B, D, F, H, J, L show the Z-linked SFS for the swan goose, mallard duck, wild turkey, helmeted guineafowl, peacock, common pheasant.

MOLECULAR ECOLOGY

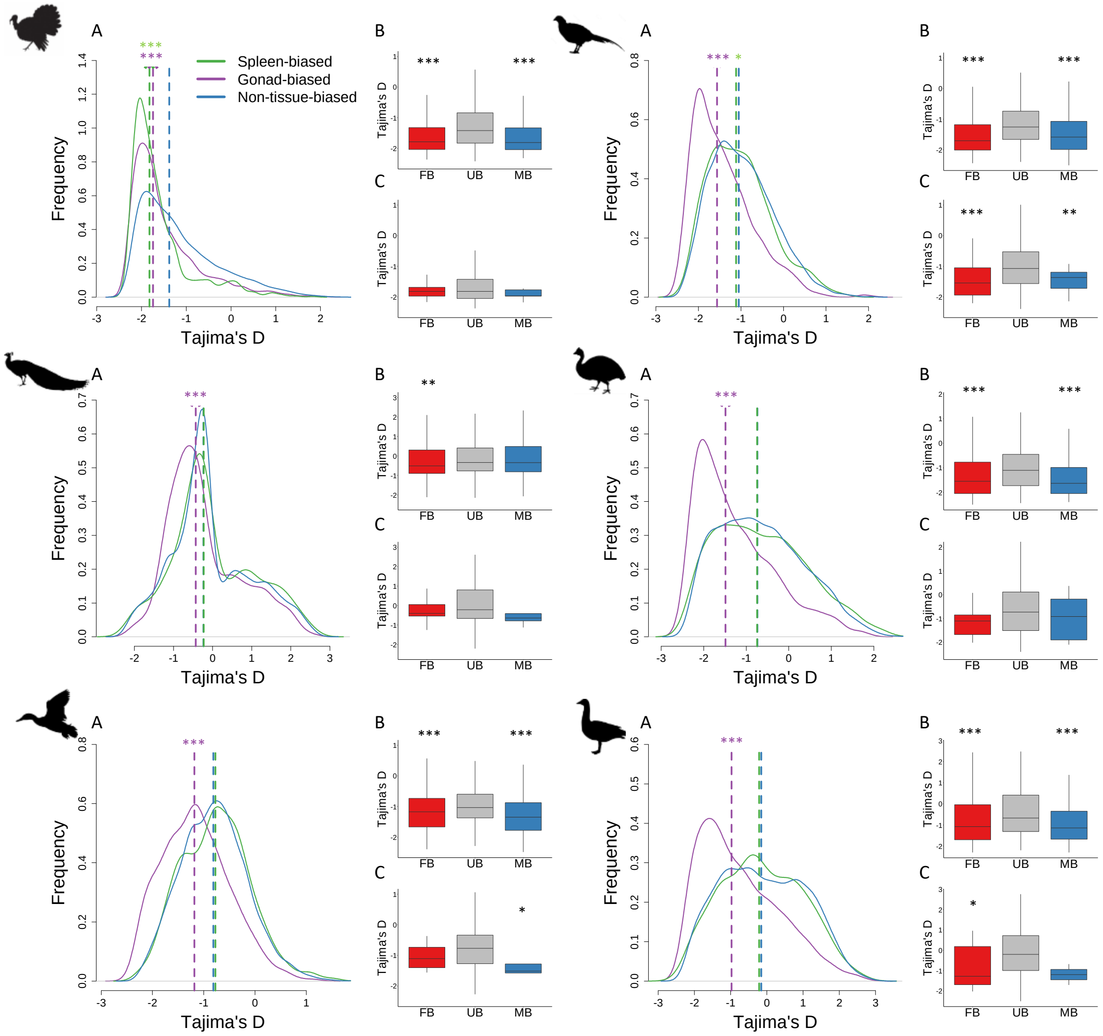

Figure S2. Patterns of Tajima's D for tissue-biased and sex-biased genes across species. Panels A show the distribution of *D* for autosomal genes for spleen-biased, gonad-biased and non-tissue-biased genes. Dotted lines show median *D* for each set of genes and *,**,*** denote a significant difference relative to non-tissue-biased genes (Wilcoxon test, $p < 0.05$, $p < 0.01$, $p < 0.001$). Tissue-biased genes were identified from female expression data. Panels B and C show the relationship between *D* and expression for genes with gonad-biased expression (panel B) or spleen-biased expression (panel C). *,**,*** denote a significant difference relative to unbiased genes (Wilcoxon test, $p < 0.05$, $p < 0.01$, $p < 0.001$). FB, UB, MB refer to female-biased, unbiased and male-biased genes respectively.

MOLECULAR ECOLOGY

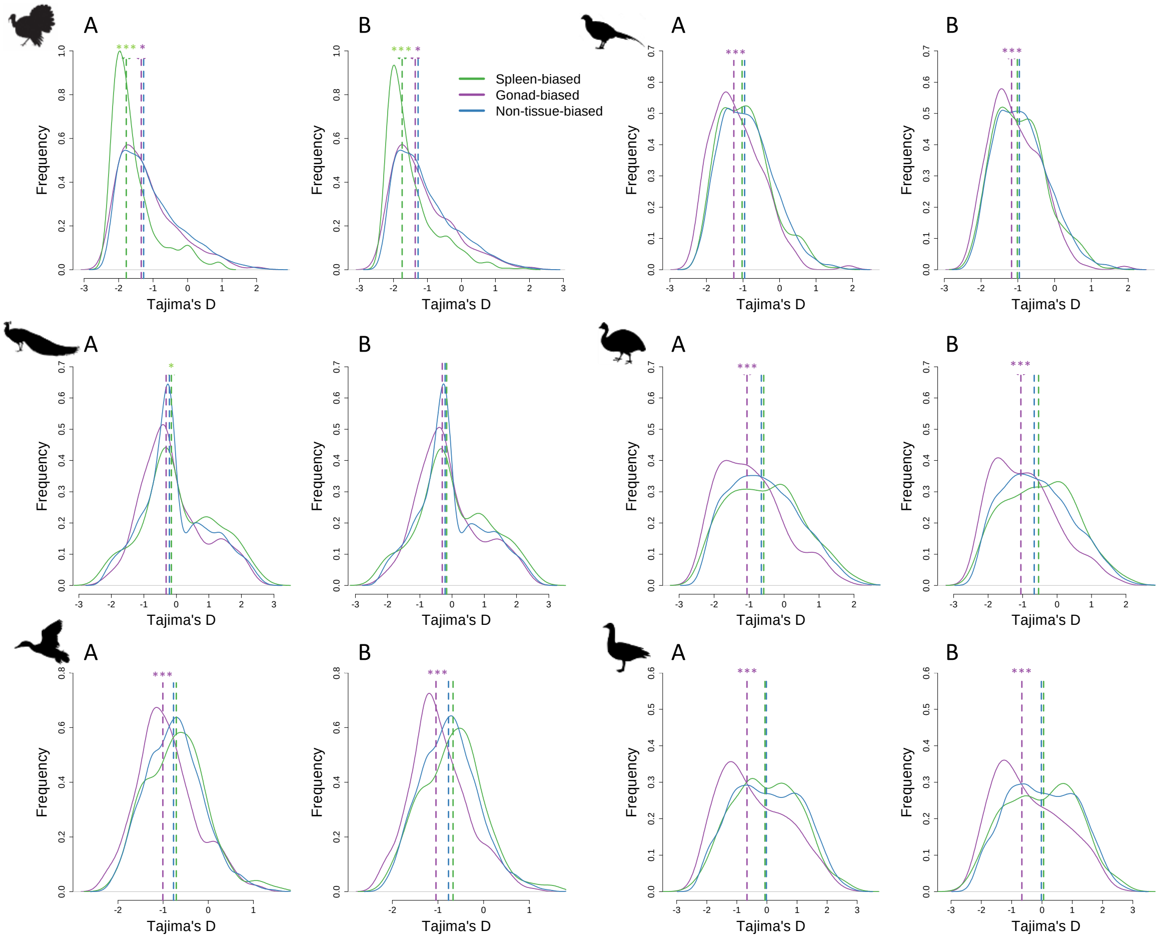

Figure S3. Distribution of Tajima's D for autosomal genes with unbiased expression between males and females that are spleen-biased, gonad-biased and non-tissue-biased in each species. Panels A show Tajima's D for tissue-biased genes identified from female expression data and panels B from male expression data. Dotted lines show median Tajima's D for each set of genes and *,**,*** denote a significant difference relative to non-tissue-biased genes (Wilcoxon test, $p < 0.05$, $p < 0.01$, $p < 0.001$).

MOLECULAR ECOLOGY

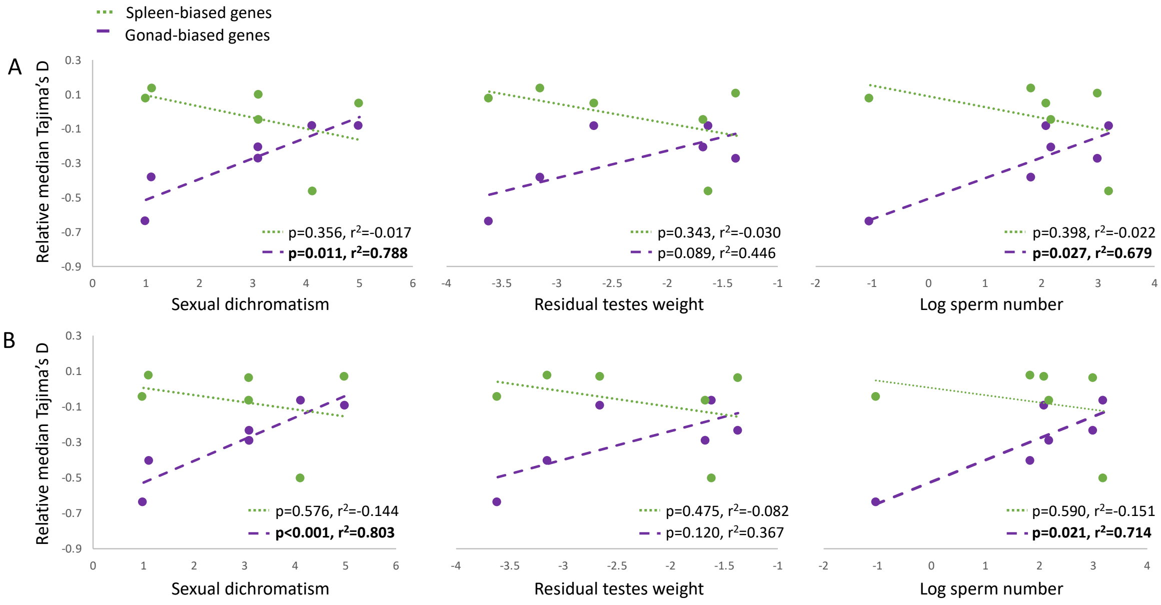

Figure S4. Phylogenetically controlled regression between proxies of sperm competition and relative Tajima's D for autosomal genes with unbiased expression between males and females. Relative *D* is calculated as the difference between median *D* for tissue-biased genes compared to non-tissue-biased genes. Tissue-biased genes were identified from male expression data (panel A) and female expression (panel B).

MOLECULAR ECOLOGY

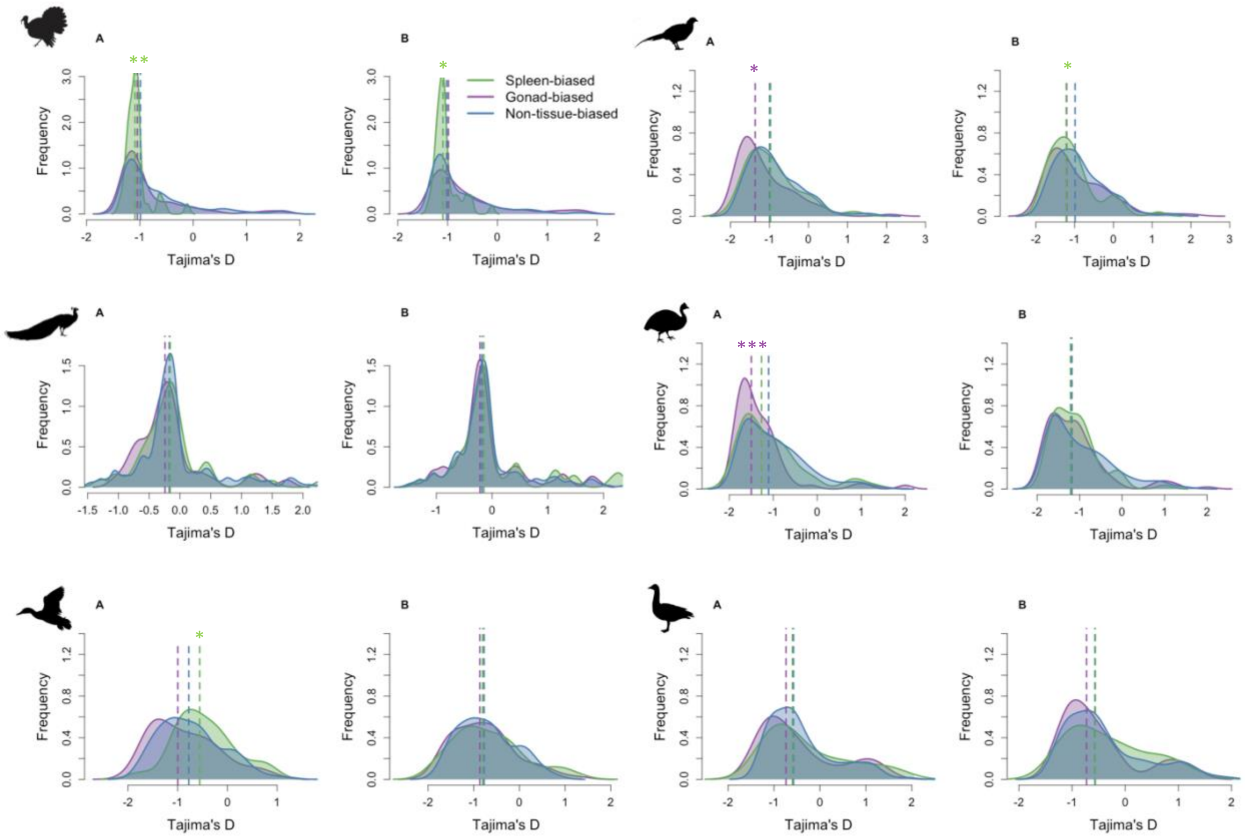

Figure S5. Distribution of Tajima's D for Z-linked genes that are spleen-biased, gonad-biased and non-tissue-biased in each species. Panels A show Tajima's D for tissue-biased genes identified from female expression data and panels B from male expression data. Dotted lines show median Tajima's D for each set of genes and *,**,*** denote a significant difference relative to non-tissue-biased genes (Wilcoxon test, $p < 0.05$, $p < 0.01$, $p < 0.001$).

MOLECULAR ECOLOGY

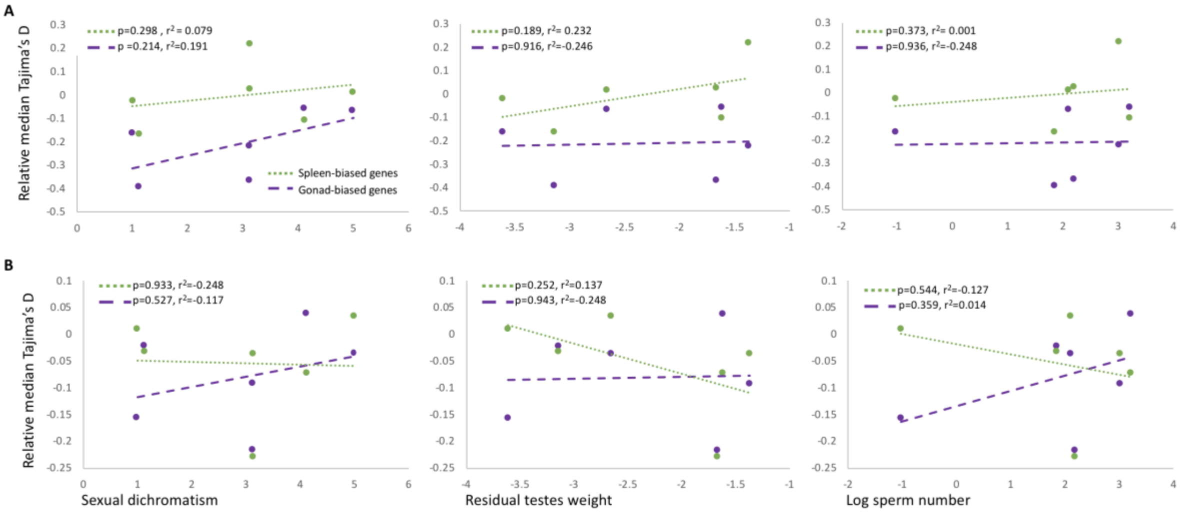

Figure S6. Phylogenetically controlled regression between proxies of sperm competition and relative Tajima's D for Z-linked genes. Relative *D* is calculated as the difference between median *D* for tissue-biased genes compared to non-tissue-biased genes. Tissue-biased genes were identified from female expression data (panels A) or male data (panels B).

MOLECULAR ECOLOGY

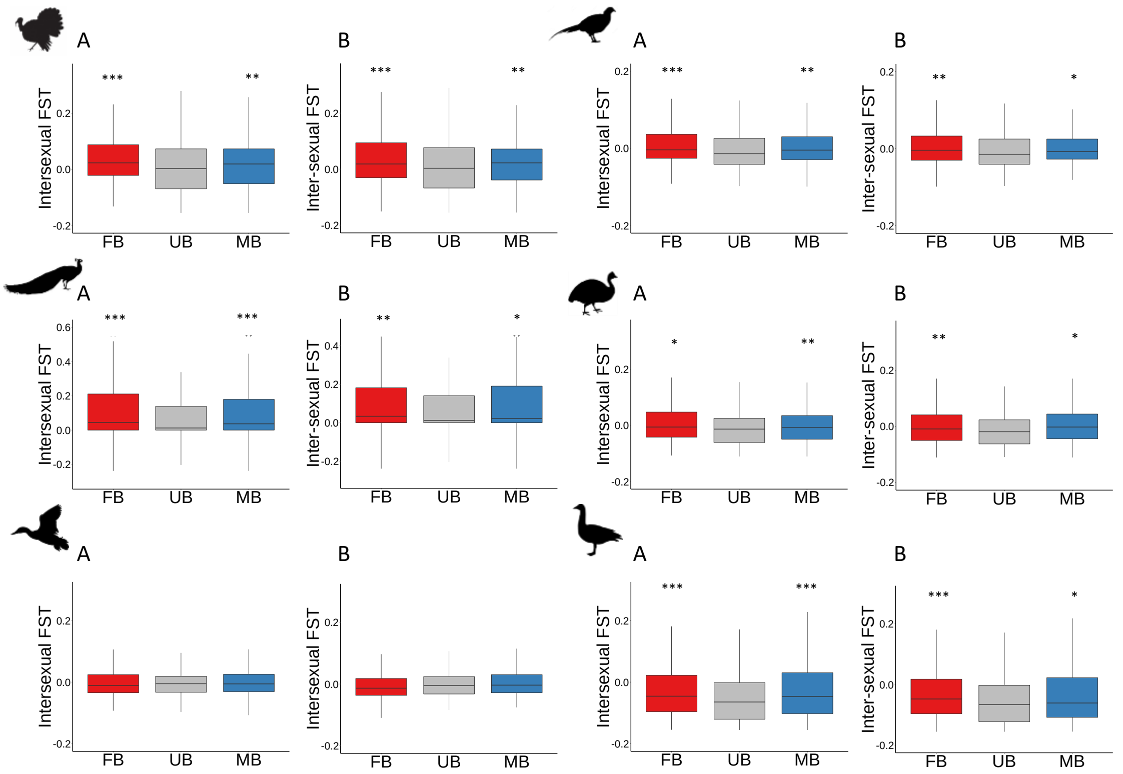

Fig S7. Patterns of inter-sexual F_{ST} for different sex-bias classes of autosomal genes expressed primarily in the gonad across various species of fowl. Panels A show F_{ST} for gonad-biased genes identified from male expression data and panels B from female expression data. *,**,*** denote a significant difference relative to unbiased genes (Wilcoxon test, $p < 0.05$, $p < 0.01$, $p < 0.001$).

MOLECULAR ECOLOGY

Table S1: Statistics of transcriptome assembly

Species	No. transcripts	No. 'best isoforms'	No. 2FPKM filtered 'best isoforms'	% Complete BUSCOs	No. pairwise reciprocal orthologs	No. autosomal/Z-linked genes
Mallard duck	1,158,494	691,200	64,017	75.6%	11,608	8,305/342
Swan goose	1,096,179	709,410	50,187	70.0%	11,479	8,180/345
Wild turkey	1,066,855	676,369	56,876	69.9%	11,454	10,285/532
Common pheasant	1,160,278	699,055	74,515	68.4%	11,558	10,328/544
Guineafowl	1,110,494	686,549	55,050	77.6%	11,419	10,233/531
Indian peafowl	1,057,022	655,810	57,425	68.4%	11,085	9,886/528

Table S2: Tissue-biased genes identified in males

	Autosomes		Z chromosome	
**Species**	**No. spleen-biased genes**	**No. gonad-biased genes**	**No. spleen-biased genes**	**No. gonad-biased genes**
Mallard duck	971	1647	43	66
Swan goose	893	1545	29	62
Wild turkey	1367	2052	44	133
Common pheasant	1236	2267	49	139
Guineafowl	1379	1964	108	61
Indian peafowl	1278	2113	53	130

MOLECULAR ECOLOGY

Table S3: Tissue-biased genes identified in females

	Autosomes		Z chromosome	
**Species**	**No. spleen-biased genes**	**No. gonad-biased genes**	**No. spleen-biased genes**	**No. gonad-biased genes**
Mallard duck	689	1283	25	48
Swan goose	651	1262	29	44
Wild turkey	890	1624	39	86
Common pheasant	774	1533	33	89
Guineafowl	1004	1446	52	70
Indian peafowl	891	1506	45	77

Table S4: Intersexual F_{ST} across autosomal tissue-biased genes

**Species**	**Gonad-biased**		**Spleen-biased**		**Non-tissue-biased**
	**Median F_{ST}**	**p-value**	**Median F_{ST}**	**p-value**	**Median F_{ST}**
Mallard duck	-0.006	0.660	-0.007	0.891	-0.007
Swan goose	-0.061	0.068	-0.064	**0.041**	-0.078
Wild turkey	0.001	0.359	0.014	**0.015**	0.001
Common pheasant	-0.014	0.311	-0.009	0.754	-0.008
Guinea fowl	-0.010	0.452	-0.020	0.181	-0.017
Indian peafowl	0.012	0.771	0.003	0.253	0.010

Only unbiased genes were used in this analysis. Tissue-biased genes were identified from male expression data. P-values are relative to non-tissue-biased genes and were calculated using Wilcoxon rank tests.

MOLECULAR ECOLOGY

Table S5: Intersexual F_{ST} across autosomal tissue-biased genes

Species	Gonad-biased		Spleen-biased		Non-tissue-biased
	Median F_{ST}	p-value	Median F_{ST}	p-value	Median F_{ST}
Mallard duck	-0.005	0.570	-0.005	0.326	-0.007
Swan goose	-0.062	0.154	-0.072	0.271	-0.077
Wild turkey	<0.001	0.384	0.012	0.126	0.001
Common pheasant	-0.014	0.547	-0.001	0.458	-0.008
Guinea fowl	-0.019	0.956	-0.020	0.181	-0.016
Indian peafowl	0.012	0.746	0.003	0.103	0.010

Only unbiased genes were used in this analysis. Tissue-biased genes were identified from female expression data. P-values are relative to non-tissue-biased genes and were calculated using Wilcoxon rank tests.

Table S6: Observed and expected number of genes with intersexual $F_{ST} > 0$ across tissue-biased genes

Species	Gonad-biased			Spleen-biased		
	E	O	p-value	E	O	p-value
Mallard duck	112	107	0.670	108	109	0.961
Swan goose	51	57	0.398	52	57	0.482
Wild turkey	171	162	0.483	172	195	0.083
Common pheasant	137	136	0.936	128	117	0.350
Guineafowl	101	104	0.786	136	124	0.323
Indian peafowl	193	202	0.530	200	189	0.433

Only unbiased genes were used in this analysis. Tissue-biased genes were identified from female expression data. Only autosomal genes are included in the analyses. Expected number of genes with intersexual $F_{ST} > 0$ were calculated from observations of F_{ST} in non-tissue-specific genes. P-values were calculated using chi-squared tests.

MOLECULAR ECOLOGY

Table S7: Model selection for relationship between Tajima's D and sex-bias for autosomal gonad-biased genes**.**

	Mallard duck		Swan goose		Wild turkey		Common pheasant		Guinea fowl		Indian peafowl	
**Model**	**Δ AIC**	**LLV p-value***	**Δ AIC**	**LLV p-value***	**Δ AIC**	**LLV p-value***	**Δ AIC**	**LLV p-value***	**Δ AIC**	**LLV p-value***	**Δ AIC**	**LLV p-value***
**Intercept**	117	-1291 p< 0.001	139	-1895 p< 0.001	181	-1852 p< 0.001	201	-1647 p< 0.001	109	-1935 p< 0.001	12	-2047 p< 0.001
**Linear**	112	-1289 p< 0.001	140	-1894 p< 0.001	179	-1851 p< 0.001	185	-1638 p< 0.001	108	-1934 p< 0.001	6	-2044 p< 0.001
**2nd ° polynomial**	22	-1242 p< 0.001	31	-1838 p< 0.001	57	-1789 p< 0.001	42	-1566 p< 0.001	30	-1894 p< 0.001	-	-2039
**3rd ° polynomial**	4	-1232 p=0.017	33	-1838 p< 0.001	56	-1787 p< 0.001	42	-1565 p< 0.001	32	-1894 p< 0.001	2	-2039 p=0.875
**4th ° polynomial**	1	-1229 p=0.071	8	-1825 p=0.002	17	-1766 p< 0.001	0	-1543 p=0.132	3	-1879 p=0.032	NA	NA
**5th ° polynomial**	0	-1228 p=0.116	10	-1825 p< 0.001	11	-1762 p< 0.001	2	-1565 p<0.05	3	-1878 p=0.027	NA	NA
**6th ° polynomial**	-	-1227	-	-1819	1	-1756 p=0.071	-	-1540	1	-1875 p=0.101	NA	NA
**7th ° polynomial**	2	-1227 p=0.606	2	-1819 p=0.718	-	-1755	2	-1540 p=0.569	-	-1874	NA	NA
**8th ° polynomial**	NA	NA	NA	NA	1	-1755 p=0.224	NA	NA	2	-1874 p=0.816	NA	NA
**Best fit adjusted r^2**	**4th ° polynomial 0.089**		**6th ° polynomial 0.109**		**6th ° polynomial 0.108**		**4th ° polynomial 0.125**		**6th ° polynomial 0.076**		**2nd ° polynomial 0.009**	

Tissue-biased expression was estimated using comparisons between female expression. *Significance test relative to the model with the lowest AIC. A significant p-value indicates the model with the lowest AIC is the better fitting model. Models within 2 AIC units of the model with the lowest AIC (or p <0.05) were treated as one top model set, and the model with the fewest parameters was preferred.

MOLECULAR ECOLOGY

Table S8: Model selection for relationship between Tajima's D and sex-bias for autosomal gonad-biased genes**.**

	Mallard duck		Swan goose		Wild turkey		Common pheasant		Guinea fowl		Indian peafowl	
**Model**	**Δ AIC**	**LLV<br>p-value***	**Δ AIC**	**LLV<br>p-value***	**Δ AIC**	**LLV<br>p-value***	**Δ AIC**	**LLV<br>p-value***	**Δ AIC**	**LLV<br>p-value***	**Δ AIC**	**LLV<br>p-value***
**Intercept**	209	-1671<br>p< 0.001	241	-2279<br>p< 0.001	251	-2371<br>p< 0.001	378	-2470<br>p< 0.001	214	-2626<br>p< 0.001	14	-2868<br>p< 0.001
**Linear**	200	-1666<br>p< 0.001	207	-2261<br>p< 0.001	216	-2353<br>p< 0.001	349	-2454<br>p< 0.001	205	-2621<br>p< 0.001	16	-2868<br>p< 0.001
**2nd ° polynomial**	50	-1590<br>p< 0.001	95	-2204<br>p< 0.001	106	-2297<br>p< 0.001	120	-2339<br>p< 0.001	59	-2547<br>p< 0.001	1	-2860<br>p=0.050
**3rd ° polynomial**	24	-1576<br>p< 0.001	48	-2179<br>p< 0.001	58	-2292<br>p< 0.001	85	-2320<br>p< 0.001	48	-2540<br>p< 0.001	1	-2858<br>p=0.085
**4th ° polynomial**	16	-1570<br>p< 0.001	30	-2170<br>p< 0.001	39	-2262<br>p< 0.001	27	-2290<br>p< 0.001	10	-2520<br>p=0.001	3	-2858<br>p=0.037
**5th ° polynomial**	8	-1566<br>p=0.001	-	-2154	12	-2247<br>p< 0.001	12	-2282<br>p< 0.001	4	-2517<br>p=0.017	1	-2657<br>p=0.090
**6th ° polynomial**	-	-1561	2	-2154<br>p=0.886	10	-2245<br>p<0.001	4	-2277<br>p=0.019	1	-2514<br>p=0.063	-	-2855
**7th ° polynomial**	2	-1560<br>p=0.527	NA	NA	-	-2239	-	-2274	-	-2512	2	-2855<br>p=0.756
**8th ° polynomial**	NA	NA	NA	NA	2	-2239<br>p=0.778	2	-2274<br>p=0.702	0	-2511<br>p=0.164	NA	NA
**Best fit<br>adjusted r^2**	**6th ° polynomial<br>0.123**		**5th ° polynomial<br>0.147**		**7th ° polynomial<br>0.118**		**7th ° polynomial<br>0.156**		**6th ° polynomial<br>0.105**		**2nd ° polynomial<br>0.007**	

Tissue-biased expression was estimated using comparisons between male expression. *Significance test relative to the model with the lowest AIC. A significant p-value indicates the model with the lowest AIC is the better fitting model. Models within 2 AIC units of the model with the lowest AIC (or p <0.05) were treated as one top model set, and the model with the fewest parameters was preferred.

MOLECULAR ECOLOGY

Table S9: Model selection for relationship between Tajima's D and sex-bias for autosomal spleen-biased genes**.**

	Mallard duck		Swan goose		Wild turkey		Common pheasant		Guinea fowl		Indian peafowl	
**Model**	**Δ AIC**	**LLV p-value***	**Δ AIC**	**LLV p-value***	**Δ AIC**	**LLV p-value***	**Δ AIC**	**LLV p-value***	**Δ AIC**	**LLV p-value***	**Δ AIC**	**LLV p-value***
**Intercept**	0	-699 p=0.094	7	-998 p=0.004	-	-923	12	-867 p<0.001	4	-1488 p=0.023	-	-1316
**Linear**	2	-699 p=0.041	5	-996 p=0.009	2	-923 p=0.888	11	-866 p<0.001	4	-1487 p=0.011	2	-1316 p=0.612
**2nd ° polynomial**	4	-699 p=0.013	1	-993 p=0.09	1	-921 p=0.183	-	-860	-	-1484	3	-1315 p=0.614
**3rd ° polynomial**	-	-696	-	-991	1	-920 p=0.165	2	-860 p=0.841	1	-1484 p=0.372	5	-1315 p=0.803
**4th ° polynomial**	2	-696 p=0.593	1	-990 p=0.308	3	-920 p=0.249	NA	NA	NA	NA	NA	NA
**Best fit adjusted r^2**	**Intercept**		**2nd ° polynomial 0.013**		**Intercept**		**2nd ° polynomial 0.018**		**2nd ° polynomial 0.005**		**Intercept**	

Tissue-biased expression was estimated using comparisons between female expression. *Significance test relative to the model with the lowest AIC. A significant p-value indicates the model with the lowest AIC is the better fitting model. Models within 2 AIC units of the model with the lowest AIC (or p <0.05) were treated as one top model set, and the model with the fewest parameters was preferred.

MOLECULAR ECOLOGY

Table S10: Model selection for relationship between Tajima's D and sex-bias for autosomal spleen-biased genes**.**

	**Mallard duck**		**Swan goose**		**Wild turkey**		**Common pheasant**		**Guinea fowl**		**Indian peafowl**	
**Model**	**Δ AIC**	**LLV p-value***	**Δ AIC**	**LLV p-value***	**Δ AIC**	**LLV p-value***	**Δ AIC**	**LLV p-value***	**Δ AIC**	**LLV p-value***	**Δ AIC**	**LLV p-value***
**Intercept**	-	-972	7	-1400<br>p=0.004	2	-1547<br>p=0.250	21	-1406<br>p<0.001	5	-2028<br>p=0.010	-	-1900
**Linear**	2	-972<br>p=0.876	5	-1398<br>p=0.009	4	-1547<br>p=0.133	22	-1406<br>p<0.001	7	-2028<br>p=0.005	2	-1900<br>p=0.846
**2nd ° polynomial**	3	-971<br>p=0.666	-	-1395	6	-1547<br>p=0.006	7	-1397<br>p=0.004	3	-2025<br>p=0.031	3	-1899<br>p=0.769
**3rd ° polynomial**	5	-9.71<br>p=0.700	2	-1395<br>p=0.967	1	-1544<br>p=0.055	7	-1396<br>p=0.005	-	-2022	5	-1899<br>p=0.857
**4th ° polynomial**	6	-971<br>p=0.797	4	-1395<br>p=0.819	1	-1543<br>p=0.095	1	-1392<br>p=0.083	2	-2022<br>p=0.870	NA	NA
**5th ° polynomial**	NA	NA	NA	NA	0	-1541<br>p=0.131	3	-1392<br>p=0.031	NA	NA	NA	NA
**6th ° polynomial**	NA	NA	NA	NA	-	-1540	-	-1390	NA	NA	NA	NA
**7th ° polynomial**	NA	NA	NA	NA	0	-1539<br>p=0.196	1	-1390<br>p=0.391	NA	NA	NA	NA
**Best fit**<br>**adjusted r^2**	**Intercept**		**2nd ° polynomial**<br>**0.010**		**Intercept**		**4th ° polynomial**<br>**0.018**		**3rd ° polynomial**<br>**0.006**		**Intercept**	

Tissue-biased expression was estimated using comparisons between male expression. *Significance test relative to the model with the lowest AIC. A significant p-value indicates the model with the lowest AIC is the better fitting model. Models within 2 AIC units of the model with the lowest AIC (or p <0.05) were treated as one top model set, and the model with the fewest parameters was preferred.

Table S11: The effect of tissue-bias on Tajima's D and F_{ST} after controlling for multiple factors using multiple regression.

	Tajima's D		F_{ST}
**Species**	**All genes p-value (F value)**	**Unbiased genes p-value (F value)**	**Unbiased genes p-value (F value)**
Mallard duck	**< 2.2e-16** (38.093)	**< 0.001** (8.525)	0.687 (0.375)
Swan goose	**< 2.2e-16** (85.478)	**3.184e-05** (10.383)	0.421 (0.866)
Wild turkey	**< 2.2e-16** (157.101)	**< 2.2e-16** (51.923)	0.498 (0.698)
Common pheasant	**< 2.2e-16** (42.168)	**0.025** (3.697)	0.509 (0.676)
Guineafowl	**< 2.2e-16** (90.883)	**9.695e-07** (13.889)	0.480 (0.734)
Indian peafowl	**0.024** (3.718)	0.114 (2.174)	0.767 (0.265)

Tissue-biased genes were identified from male expression data. Only autosomal genes are included in the analyses. Multi-predictor model: (TD/F_{ST} ~ Tissue bias + log(tW) + log(Gene length) + log(GC) + log (Gene expression level)).

MOLECULAR ECOLOGY

Table S12: The effect of sex-bias on Tajima's D and F_{ST} after controlling for multiple factors using multiple regression.

**Species**	**Tajima's D p-value (F value)**	**F_{ST} p-value (F value)**
Mallard duck	**0.029** (3.560)	0.597 (0.516)
Swan goose	**<0.001** (7.432)	**0.008** (4.905)
Wild turkey	**8.254e-07** (14.153)	**0.048** (3.053)
Common pheasant	**1.026e-05** (42.168)	0.083 (2.489)
Guineafowl	0.076 (11.585)	0.568 (0.567)
Indian peafowl	0.884 (0.123)	0.099 (2.316)

Only autosomal genes with gonad-biased expression are included in the analyses. Tissue-biased genes were identified from male expression data. Multi-predictor model: (TD/F_{ST} ~ Sex bias + log(tW) + log(Gene length) + log(GC) + log (Gene expression level)).

REFERENCES

Burnham KP, Anderson DR. 2003. Model selection and multimodel inference: a practical information-theoretic approach: Springer Science & Business Media, Berlin, Germany.

Cheng C, Kirkpatrick M. 2016. Sex-specific selection and sex-biased gene expression in humans and flies. PLOS Genetics 12:e1006170.

Drummond AJ, Suchard MA, Xie D, Rambaut A. 2012. Bayesian Phylogenetics with BEAUti and the BEAST 1.7. Molecular Biology and Evolution 29:1969-1973.

Fox E, Wright A, Fumagalli M, Vieira F. 2019. ngsLD: evaluating linkage disequilibrium using genotype likelihoods. Bioinformatics

Fumagalli M, Vieira FG, Linderoth T, Nielsen R. 2014. ngsTools: methods for population genetics analyses from next-generation sequencing data. Bioinformatics 30:1486-1487.

Hackett SJ, Kimball RT, Reddy S, Bowie RCK, Braun EL, Braun MJ, Chojnowski JL, Cox WA, Han K-L, Harshman J, et al. 2008. A phylogenomic study of birds reveals their evolutionary history. Science 320:1763-1768.

Harrison PW, Wright AE, Zimmer F, Dean R, Montgomery SH, Pointer MA, Mank JE. 2015. Sexual selection drives evolution and rapid turnover of male gene expression. Proceedings of the National Academy of Sciences 112:4393-4398.

Jetz W, Thomas GH, Joy JB, Hartmann K, Mooers AO. 2012. The global diversity of birds in space and time. Nature 491:444-448.

Korneliussen TS, Moltke I. 2015. NgsRelate: a software tool for estimating pairwise relatedness from next-generation sequencing data. Bioinformatics 31:4009-4011.

Paradis E, Claude J, Strimmer K. 2004. APE: Analyses of phylogenetics and evolution in R language. Bioinformatics 20:289-290.

Team RC. 2016. R: A language and environment for statistical computing. R Foundation for Statistical Computing, Vienna, Austria.

Wright AE, Fumagalli M, Cooney CR, Bloch NI, Vieira FG, Buechel SD, Kolm N, Mank JE. 2018. Male-biased gene expression resolves sexual conflict through the evolution of sex-specific genetic architecture. Evolution Letters 2:52-61.

Zeileis A, Hothorn T. 2002. Diagnostic checking in regression relationships. R News.

MOLECULAR ECOLOGY
